# Research progress of vanadium pentoxide photocatalytic materials

**DOI:** 10.1039/d3ra03648k

**Published:** 2023-07-31

**Authors:** Yanlin Li, Shenghua Chen, Wenyuan Duan, Yanli Nan, Donghai Ding, Guoqing Xiao

**Affiliations:** a School of Materials Science and Engineering, Xi'an University of Architecture & Technology Xi'an 710055 China liyanlin@xauat.edu.cn nanyl@xauat.edu.cn; b Xi'an Key Laboratory of Advanced Photo-electronics Materials and Energy Conversion Device, Xijing University Xi'an 710123 China

## Abstract

Photocatalytic reactions convert solar energy into chemical energy through a clean and green reaction process. Photocatalytic technology based on semiconductor materials provides us with a new idea in energy utilization and environmental governance. It was found that vanadium pentoxide (V_2_O_5_) has a narrow band gap, wide response range in the visible region, high oxygen density in the V_2_O_5_ lattice, high oxidation state of V^5+^, small energy requirement, and superior catalytic activity in partial oxidation. Therefore, the utilization rate of sunlight and photocatalytic oxidation can be greatly improved using V_2_O_5_ materials. However, the narrow band gap of V_2_O_5_ also makes it easier for the photogenerated electrons and holes to recombine in the excited state, and the stored energy is instantly consumed by carrier recombination. Therefore, how to promote the carrier separation of V_2_O_5_ and improve the photocatalytic efficiency are the key problems to be solved. Herein, several methods to improve the photocatalytic performance of V_2_O_5_ are reviewed, including metallic ion doping, non-metallic ion doping, semiconductor recombination, and noble metal deposition. Finally, it is suggested that future research directions should focus on a variety of modification methods simultaneously to promote photocatalytic efficiency and lower the cost, which will enable V_2_O_5_ to have a broad development prospect in the field of photocatalysis.

## Abstract 2

Photocatalytic reactions convert solar energy into chemical energy through a clean and green reaction process.

## Graphical abstract

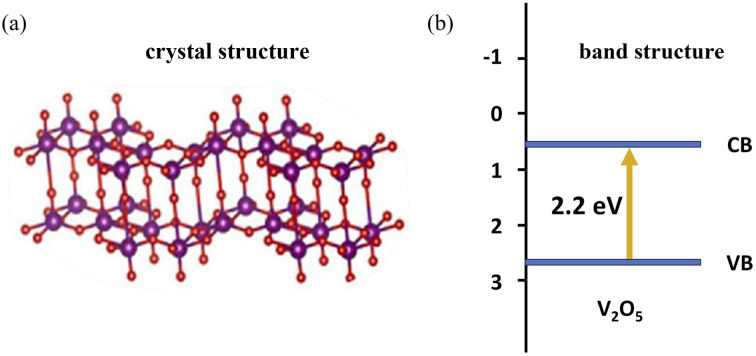

## Introduction

At present, owing to the rapid industrial development, energy and environment problems are becoming increasingly severe.^1^ The construction of an efficient, clean, low carbon and recycling green manufacturing system is put forward as one of the main strategic tasks to improve the efficiency of resource utilization and promote the efficient recycling of resources. Notably, the photocatalytic technology based on semiconductor materials provides us with a new way of thinking in energy utilization and environmental governance.

**Fig. 1:**
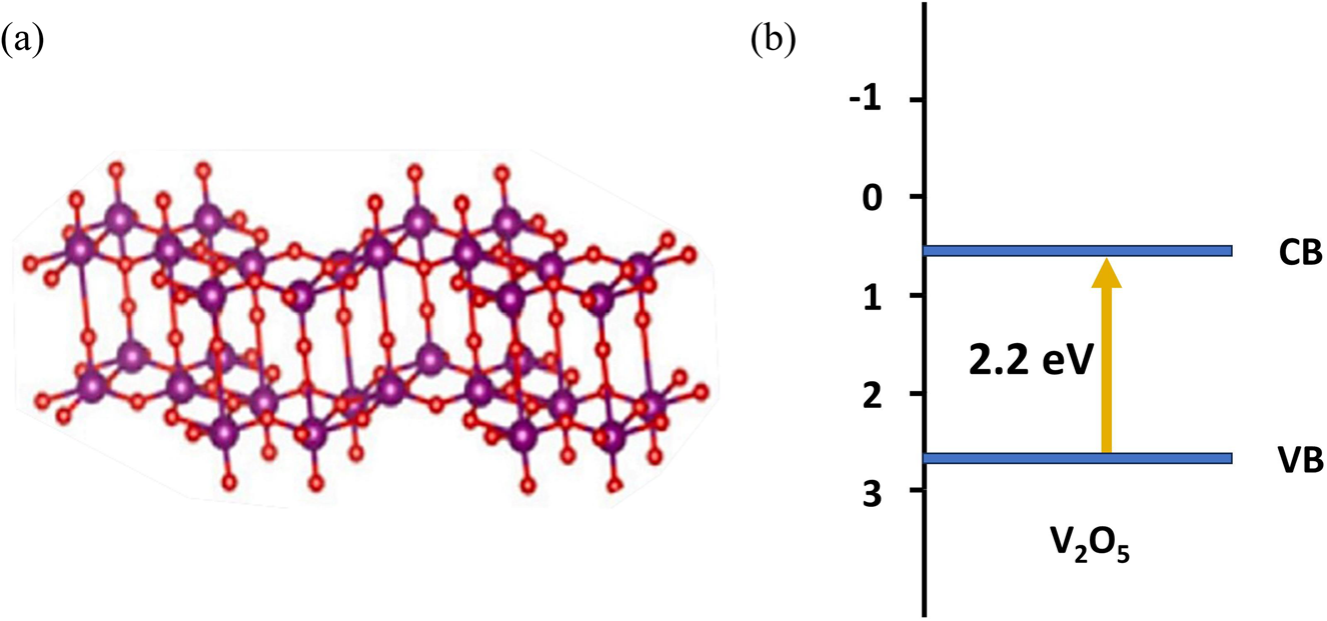
(a) The crystal structure and (b) band structure of V_2_O_5_.

There are many excellent semiconductors in the literature dealing with the photocatalytic performance, especially for transition metal oxides. Among them, due to its excellent performance, V_2_O_5_ as a highly ordered two-dimensional layered oxide^2^ has aroused great interest for a variety of important applications, such as lithium batteries, medicine, and nonferrous metal processing. It was found that V_2_O_5_ has a narrow band gap (2.2 eV), requires little energy, and shows superior catalytic activity in partial oxidation.^3–7^ The crystal structure and band structure of V_2_O_5_ are shown in Fig. 1. Therefore, photocatalysis by V_2_O_5_ materials can greatly improve the utilization rate of sunlight. Initial research studies were performed on V_2_O_5_ photocatalytic raw material mainly extracted from vanadium ore directly, but its photocatalytic performance was low. With the continuous development of nanoscience and preparation technology, researchers worldwide are actively exploring to obtain V_2_O_5_ materials by means of hydrothermal/solvothermal method, sol–gel method, electrodeposition, and high-temperature calcination to study the photocatalytic degradation and other properties of V_2_O_5_. For example, flower-like V_2_O_5_ microspheres with a diameter of ∼3 μm were synthesized by a facile hydrothermal method using ammonium metavanadate as a vanadium source, and the as-prepared V_2_O_5_ microspheres had a large surface area.^8^ Song *et al.* developed mesoporous V_2_O_5_ nanosheets by a simple hydrothermal method and subsequent instantaneous heating and calcination.^9^ The V_2_O_5_ nanosheets had a unique mesoporous nanostructure containing oxygen vacancies. A novel hierarchical starfish-like vanadium oxide was also synthesized by a direct hydrothermal method using a functional V_2_O_5_ sol as a vanadium source. Results demonstrated that the V_2_O_5_ material was composed of single crystals of a metastable VO_2_ (B) phase, which grew along the (110) plane, and four oblique sheets, which grew on the flanks of the (110) plane.^10^ Then, the starfish-like structure could be obtained after phase transition to the orthorhombic V_2_O_5_ phase while sintering at 350 °C. V_2_O_5_ materials could be also synthesized *via* a sol–gel method, which was confirmed by Yang *et al.* to fabricate the V_2_O_5_ nanoribbon.^11^ Le *et al.* reported a kind of micro–nano structural V_2_O_5_ films, which were constructed by an electrodeposition method using an aqueous solution of NH_4_VO_3_.^12^ The results showed that the surface morphology, crystal structure, and properties strongly relied on the annealing temperature. Furthermore, the excessive temperature was more likely to collapse α-V_2_O_5_ structures, which inevitably introduced a lot of defects. An interesting approach was described by Wahab *et al.* wherein a novel black V_2_O_5_ material was synthesized by a controllable and environmentally friendly physicochemical reduction method using NH_4_VO_3_ in an alumina crucible with heating at 550 °C. Furthermore, DFT analysis results revealed that tuning a high degree of surface oxygen vacancies considerably promoted the visible light photoactivity of practically inactive pristine V_2_O_5_.^13^

As a transition metal oxide of high oxidation state, V_2_O_5_ has quite a few advantages of narrow band gap, high oxygen density, high decomposition temperature, high temperature resistance, low cost, good chemical and optical stability. It shows much potential to be an excellent choice of photocatalyst.^14–16^

## Photocatalytic reaction mechanism

In terms of semiconductors, all valence electrons reside in the valence band. The band with higher energy than the valence band is the conduction band, while the gap between the valence band and the conduction band is called the forbidden band. Thus, the photocatalytic oxidation process is simply described as electrons in the valence band being excited by light and then jumping to the conduction band to form electron–hole pairs, which trigger a series of other reactions.

The photocatalytic reaction process of V_2_O_5_ mainly includes three basic steps: firstly, when light with energy greater than the band gap width (*E*_g_) is used to irradiate the photocatalyst, the electrons in the valence band of the photocatalyst are excited and jump over the band gap into the conduction band, generating electron e^−^ in the conduction band and leaving positive hole h^+^ in the valence band; secondly, the photogenerated hole h^+^ with strong oxidizing property and the photogenerated electron e^−^ with strong reducing property combine to form a redox system; finally, when the photogenerated electron–hole migrates to the surface, e^−^ can reduce the electron acceptor adsorbed on the catalyst surface, while h^+^ can combine with the electron donor adsorbed on the catalyst surface to oxidize the substances.

The specific principle of the photocatalytic reaction of V_2_O_5_ is as follows:

1 e^−^ + O_2_ → ˙O_2_^−^

2 e^−^ + R → R^−^

3 h^+^ + H_2_O → ˙OH + H^+^

4 h^+^ + OH^−^ → ˙OH

5 h^+^ + R → R^+^

6 ˙O_2_^−^ + H^+^ → ˙HO_2_

7 ˙O_2_^−^ + H_2_O → ˙OOH + OH^−^

8 2˙OOH → O_2_ + H_2_O_2_

9 H_2_O_2_ + e^−^ → ˙OH + OH^−^

10 2˙HO_2_ → O_2_ + H_2_O_2_

11 H_2_O_2_ + ˙O_2_^−^ → ˙OH + OH^−^ + O_2_

12 H_2_O_2_ + *hv* → ˙OH

13 R (R^−^, R^+^) + ˙OH → CO_2_ + H_2_O

However, the narrow band gap of V_2_O_5_ makes it easier for the photogenerated electrons and holes to recombine in the excited state, and the stored energy is instantly consumed due to carrier recombination. This recombination process occurs both on the surface and inside of the photocatalyst. After the activated e^−^ recombines with h^+^, it will dissipate its energy in the form of radiation. Therefore, how to promote the carrier separation and improve the photocatalytic efficiency of V_2_O_5_ have become the critical problems to be addressed.

14 V_2_O_5_ (h^+^) + V_2_O_5_ (e^−^) → energy

## Modification methods

There are several ways to improve the photocatalytic efficiency of V_2_O_5_ semiconductors, such as metallic ion doping, non-metallic ion doping, semiconductor recombination and noble metal deposition.

### Metallic ion doping

Electron–hole capture and recombination are two competing processes that have great influence on photocatalytic oxidation. If there is no appropriate electron and hole trapping agent in the semiconductor catalyst, the photogenerated electrons and holes will recombine inside or on the surface of the semiconductor and release energy. The recombination process can be inhibited to a certain extent by generating appropriate defects in the catalyst or introducing appropriate impurity ions as electron–hole trapping agents. Studies have shown that Fe, Co, Ni, Cu, Pt, Cr, V, Ru, Y and other metal ions doped into the semiconductor lattice can change the phase transition temperature, and then affect the photocatalytic activity.

**Fig. 2:**
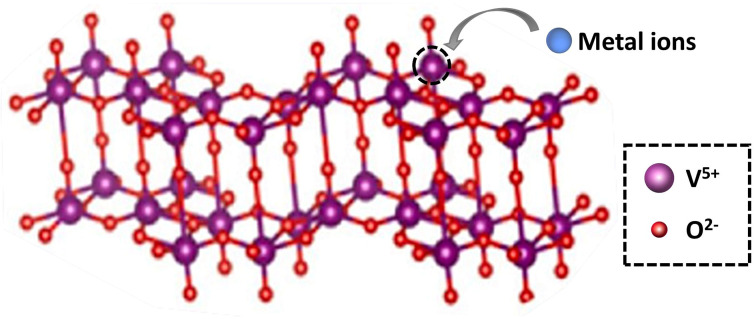
The scheme of metallic ion doping into the lattice of V_2_O_5_.

**Fig. 3:**
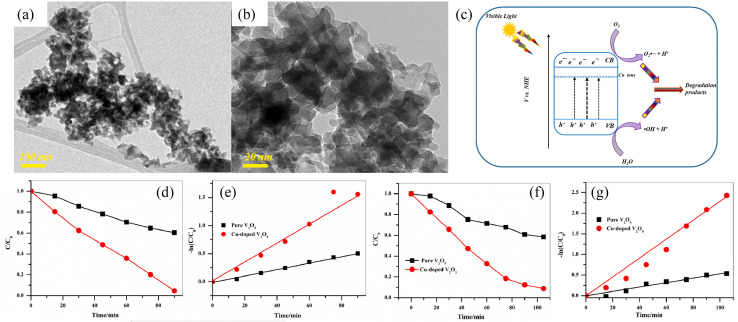
(a) TEM of V_2_O_5_, (b) TEM of Cu-doped V_2_O_5_, (c) mechanism of photocatalytic activity, (d and e) photocatalytic degradation of MB, (f and g) photocatalytic degradation of RhB (reproduced from ref. 18 with permission from [Elsevier], copyright [2023]).

**Fig. 4:**
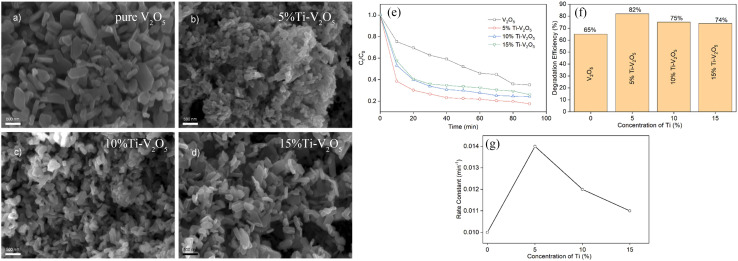
(a–d) SEM of V_2_O_5_ and Ti-doped V_2_O_5_, (e–g) photocatalytic degradation of MB (reproduced from ref. 19 with permission from [Elsevier], copyright [2022]).

The ideal doping is a metal ion with an ionic radius close to the V^5+^ radius so that the doped metal ions can effectively enter the V_2_O_5_ lattice (Fig. 2). By doping metal ions into the V_2_O_5_ lattice, new impurity levels can be introduced to broaden the response range of V_2_O_5_ to sunlight and effectively inhibit the rapid recombination of charge carriers in V_2_O_5_ materials. For example, Fe^3+^ has a similar radius to V^5+^ and easily enters the V_2_O_5_ lattice.^17^ The transition metal ions Cu-doped V_2_O_5_ nanosheets were investigated for their photocatalytic efficiency using organic dye (methyl blue (MB) and rhodamine B (RhB)) under light illumination.^18^ The results (Fig. 3) showed that Cu incorporation into the host V_2_O_5_ matrix improved the photodegradation efficiency for MB and RhB pollutants under visible light irradiation, which was attributed to the dopant ions that could separate the photoinduced electron–hole pairs and accelerate the charge movement. However, the radius of Cu^2+^ differs greatly from that of V^5+^, so the photocatalytic efficiency cannot be improved significantly by incorporation of metallic Cu. Neelima *et al.* investigated the visible-light-induced photocatalytic activity of V_2_O_5_ nanostructures doped with various amounts of Ti (Fig. 4). The radius of Ti^4+^ was close to that of V^5+^. It demonstrated that the Ti^4+^ introduced into the V_2_O_5_ lattice formed trap states within the forbidden gap. The Ti-doped V_2_O_5_ nanostructure exhibited a lower recombination rate than pure V_2_O_5_ and enhanced the photocatalytic activity.^19^ The improvement of the photocatalytic efficiency of Ti-doped V_2_O_5_ was higher than that of Cu-doped V_2_O_5_.

The choice of doped metal ions is an important factor. Suvijak *et al.* studied the effect of multiple metal ion doping on photocatalysis, such as Nb, La, Y, and Zr. They found that V_2_O_5_ modification by Nb or La dopants, which had the extra valence cations or larger ionic radius, suppressed the oxygen vacancies formation, leading to lower activity.^20^ Meanwhile, Y and Zr exhibited good capability of H_2_S removal, and the amount of surface oxygen and H_2_S conversion of the Y-doped V_2_O_5_ were more than that of the Zr-doped V_2_O_5_.

**Fig. 5:**
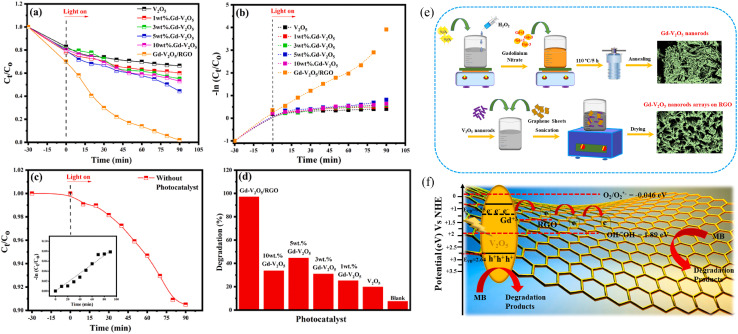
(a–d) Photocatalytic degradation of MB, (e) schematic representation of the synthesis of Gd–V_2_O_5_, (f) mechanism of photocatalytic activity (reproduced from ref. 21 with permission from [Elsevier], copyright [2021]).

**Fig. 6:**
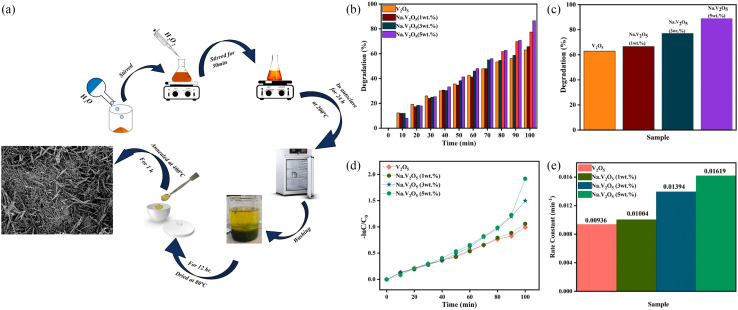
(a) Schematic representation of the synthesis of Na–V_2_O_5_, (b–e) photocatalytic degradation of RhB (reproduced from ref. 22 with permission from [Elsevier], copyright [2022]).

**Fig. 7:**
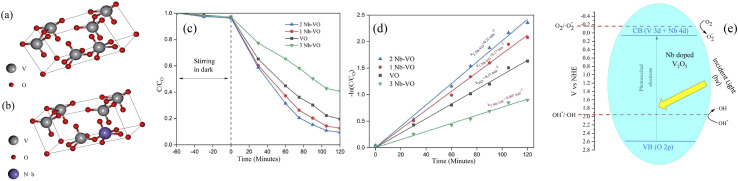
Optimized geometries of (a) the V_2_O_5_ unit crystal and (b) the Nb-doped V_2_O_5_ crystal, (c and d) photocatalytic degradation of caffeine, (e) mechanism of photocatalytic activity (reproduced from ref. 23 with permission from [RSC], copyright [2022]).

The concentration of metal ions is another important factor affecting the photocatalytic performance. Studies have shown that a lower concentration of metal ions entering the semiconductor lattice can appropriately improve its photocatalytic ability. However, the high concentration of metal ions located at the lattice site would become the carrier recombination center, which inhibited the photocatalytic activity. In addition, if the doped metal ion concentration was too high, it was easier to form a thick layer of space charge on the surface, and to a certain extent, hinder the photon absorption, thus reducing the catalytic activity. For example, a series of Gd-doped V_2_O_5_ (1 wt%, 3 wt%, 5 wt% and 10 wt%) photocatalysts had been prepared through a facile wet chemical approach, as shown in Fig. 5. The results demonstrated that 5 wt% Gd–V_2_O_5_ nanorod was in favor of MB photodegradation, and this optimum concentration probably occurred due to the quantum tunneling effect.^21^ Shahid *et al.* reported on Na-doped V_2_O_5_ nanorods for visible light-irradiated photocatalytic performance for the degradation of rhodamine dye, as shown in Fig. 6, and found that 5 wt% Na-doped V_2_O_5_ exhibited the maximum degradation with 88.9% degradation rate.^22^ Basu *et al.* found that the 2 mol% Nb-doped V_2_O_5_ nanorods achieved the maximum photocatalytic degradation of complex organic caffeine and removed 91% in 2 hours (Fig. 7).^23^

Given the above results, doping metal ions into the V_2_O_5_ lattice can introduce new impurity levels, broaden the response range of V_2_O_5_ to sunlight, and effectively inhibit the rapid recombination of electrons and holes in the V_2_O_5_ semiconductor.

### Non-metallic ion doping

Non-metallic ion doping is also confirmed to be an effective method to improve the separation efficiency of photogenerated electron–hole and inhibit the recombination of electron–hole pairs, thus promoting the quantum efficiency and catalytic activity by visible light. There are two theories in terms of non-metallic ion doping for improving photocatalytic properties. One theory states that the non-metallic elements enter the crystal lattice of the V_2_O_5_ photocatalyst. This leaves a lot of oxygen vacant defects in the forbidden band, which drive the photogenerated electrons to jump into the conduction band in two steps, resulting in it absorbing more visible light. The other theory states that non-metallic ions replace oxygen to form the structure of X–V–O–V, and then form a gap in the crystal lattice of V_2_O_5_. This introduces a new hybrid state between the conduction band and valence band to reduce the band gap width, so that the V_2_O_5_ photocatalyst has catalytic activity under visible light and improves the catalytic performance. Studies have shown that the incorporation of B, C, N, S, F, Cl and other non-metallic ions can improve the photocatalytic activity.

**Fig. 8:**
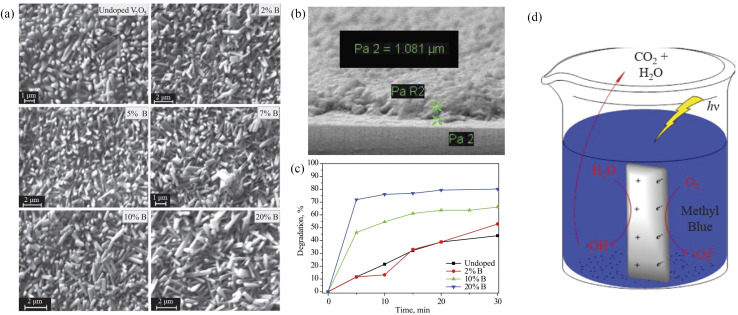
(a) SEM of V_2_O_5_ and B-doped V_2_O_5_, (b) SEM of B-doped V_2_O_5_ thin film, (c) photocatalytic degradation of MB, (d) mechanism of photocatalytic activity (reproduced from ref. 24 with permission from [Springer], copyright [2019]).

For example, the boron-doped V_2_O_5_ thin films fabricated by a spray pyrolysis technique had a large surface area. The photocatalytic degradation of methyl blue was measured in approximately 30 min in water samples under xenon light.^24^ Because of the smaller ionic radius of B^3+^ compared to that of V^5+^, the presence of boron on the surface, grain boundaries or V_2_O_5_ matrix could easily be integrated into the V_2_O_5_ skeleton without changing the initial morphology of V_2_O_5_. B-doped V_2_O_5_ was more uniform and compact than the undoped structure. This reduced the band gap width of V_2_O_5_ and enhanced the absorption of the UV and visible regions, which may be caused by the decrease of band gap energy and the increase of surface area (Fig. 8).

**Fig. 9:**
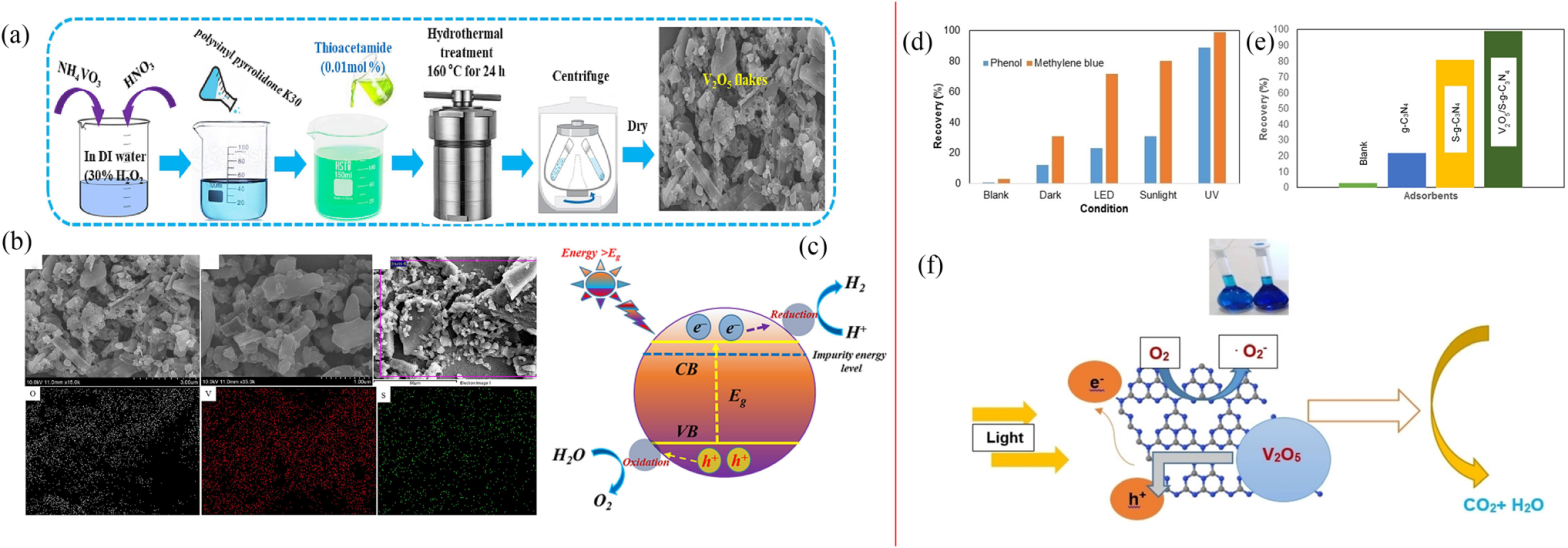
(a) Schematic representation of the synthesis of S-doped V_2_O_5_, (b) SEM of S-doped V_2_O_5_, (c) mechanism of photocatalytic activity (reproduced from ref. 25 with permission from [Elsevier], copyright [2023]). (d and e) Photocatalytic degradation of phenol and MB, (f) mechanism of photocatalytic degradation (reproduced from ref. 26 with permission from [Wiley], copyright [2019]).

Shim *et al.* studied 2D S-doped V_2_O_5_ flakes synthesized *via* a facile hydrothermal procedure (Fig. 9a). The doping of S into the host V_2_O_5_ matrix caused a positive influence on its structure, electrical conductivity and ion diffusion, and showed about 4.7 folds improvement in photocurrent density and reduced charge resistance compared to the pure V_2_O_5_.^25^ Sulfur has a larger atomic radius. Thus, doping sulfur at the oxygen site was supposed to significantly change the electronic structure of V_2_O_5_. The structural oxygen vacancies and lattice defects of S-doped V_2_O_5_ were used as carrier capture centers to reduce the photoinduced electron–hole recombination rate. In addition, S doping narrowed the band gap energy of V_2_O_5_, redshifted the absorption edge, and enhanced the visible light catalytic activity. Chegeni *et al.* also studied the S-doped V_2_O_5_ nanocomposites, which showed highly efficient degradation and adsorption for methylene blue and phenol,^26^ as shown in Fig. 9b. However, the sulfur ions were beneficial for changing the electron structure. It was difficult to replace the oxygen ions in the lattice of V_2_O_5_ because of the larger radius of sulfur ions. As a result, S-doped modification requires further study. Furthermore, we summarized the doping effects of metallic ion doping and non-metallic ion doping from references, which are shown in Table 1.

**Table 1:** The results of the doping effect in the literature

Doping elements	Results	Ref.
Cu	Improved photodegradation efficiency for MB and RhB	15
Ti	Improvement of photocatalytic efficiency of Ti–V_2_O_5_ that is higher than that of Cu–V_2_O_5_	16
Nb, La	Lower activity	17
Y, Zr	Good capability of H_2_S removal	17
Gd	Favors photodegradation of MB	18
Na	Maximum degradation with 88.9% degradation rate to RhB	19
Nb	Removed 91% organic caffeine in 2 hours	20
B	Photodegradation for MB within 30 min in water samples under xenon light	21
S	4.7-Fold improvement in photocurrent density	22
S	Improved photodegradation for MB and phenol	23

### Semiconductor recombination

**Fig. 10:**
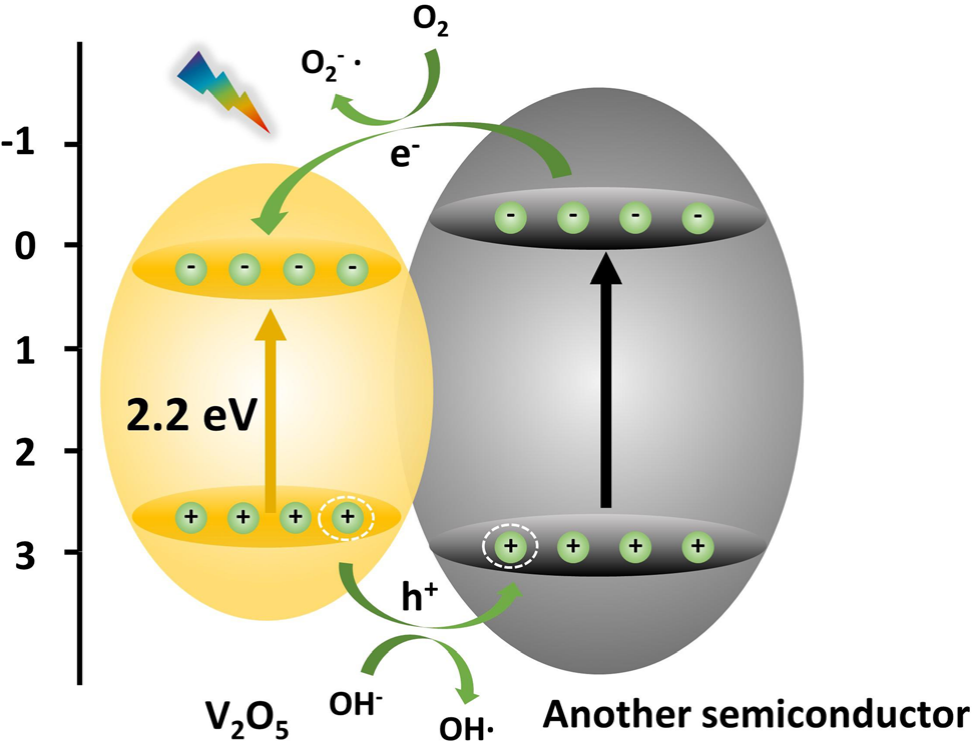
The scheme of the band structure of V_2_O_5_ and other semiconductor composites.

In order to promote carrier separation, the semiconductors with different band structures were combined. Thus, the wide-band gap photocatalysts were sensitized with narrow-band gap semiconductors to improve the photocatalytic performance. By combining the V_2_O_5_ semiconductor with materials with different bandgap widths, the composite semiconductors were prepared, so as to effectively improve the utilization efficiency of visible light of the V_2_O_5_ composite materials (Fig. 10). Different oxide or sulfide semiconductors have different effects on the photocatalytic performance of composites. According to literature reports, the optimal band gap of the actual photocatalyst is about 1.8 eV. Therefore, the reasonable selection of composite is very important to improve the photocatalytic performance of V_2_O_5_.

The interfacial transfer rate of electrons and holes is much slower than that of the capture or recombination process of photogenerated charge. If the interfacial transfer rate of electrons and holes can be accelerated to reduce the accumulation of photogenerated charge in the semiconductor, the recombination probability of photogenerated electrons and holes can also be reduced and the efficiency of photocatalytic oxidation can be improved. The band gap of the V_2_O_5_ semiconductor is relatively narrow, and the photogenerated electrons and holes generated in the photocatalytic reaction are easy to recombine. However, the band structure and band gap width of different conductors are different. Therefore, the appropriate semiconductor can be selected to combine with V_2_O_5_. The characteristics of the band structure of the two semiconductors can be used to accelerate the interface transfer rate of electrons and holes, and improve the photocatalytic efficiency.

**Fig. 11:**
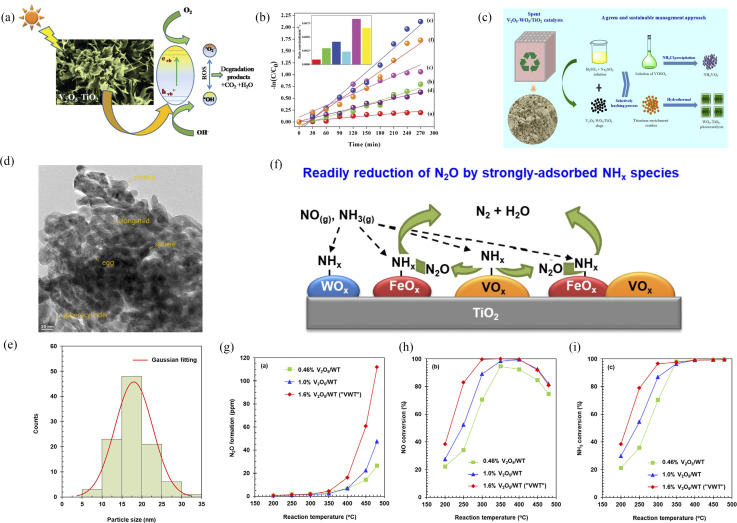
(a and b) Schematic photocatalytic degradation and photocatalytic properties of V_2_O_5_–TiO_2_ (reproduced from ref. 27 with permission from [RSC], copyright [2019]). (c) Schematic synthesis of V_2_O_5_–WO_3_/TiO_2_ (reproduced from ref. 28 with permission from [ACS], copyright [2018]). (d–i) Formation and depression of N_2_O in the selective reduction of NO by NH_3_ over V_2_O_5_-WO_3_/TiO_2_ catalysts (reproduced from ref. 29 with permission from [Elsevier], copyright [2021]).

Typically, when sunlight irradiates the V_2_O_5_–TiO_2_ composites, the electrons in the V_2_O_5_ valence band are first excited and jump to the conduction band, where e^−^ is produced and h^+^ is left in the valence band. TiO_2_ is then activated to perform the same photogeneration carrier process inside the semiconductor. Due to the difference in their potential, h^+^ in the V_2_O_5_ valence band flows to the TiO_2_ valence band, while e^−^ in the TiO_2_ conduction band flows to the V_2_O_5_ conduction band. Thus, photogenerated electrons and holes can be effectively separated, and the accumulation of photogenerated charges in semiconductor can be reduced. Furthermore, the recombination probability of photogenerated electrons and holes can be reduced, and the efficiency of photocatalytic oxidation can be improved. In Fig. 11, V_2_O_5_–TiO_2_ nanocomposites were synthesized by a one-step mechanical alloying method. The photocatalytic degradation of rhodamine B under visible light had been enhanced significantly in comparison with both nanocrystalline TiO_2_ and V_2_O_5_.^27^

One semiconductor composite with only TiO_2_ modification has a limit for improvement of photocatalytic performance. Furthermore, corrosion-resistant and stable WO_3_ materials have attracted much attention. Wu *et al.* developed an efficient and sustainable process for the recovery of vanadium and produced V_2_O_5_–WO_3_/TiO_2_ photocatalytic materials.^28^ Nguyen *et al.* also reported the formation and depression of N_2_O in the selective reduction of NO by NH_3_ over V_2_O_5_-WO_3_/TiO_2_ catalysts.^29^ As a result, the use of both WO_3_ and TiO_2_ was more effective for improving photocatalytic performance than that with only TiO_2_ or pure V_2_O_5_.

**Fig. 12:**
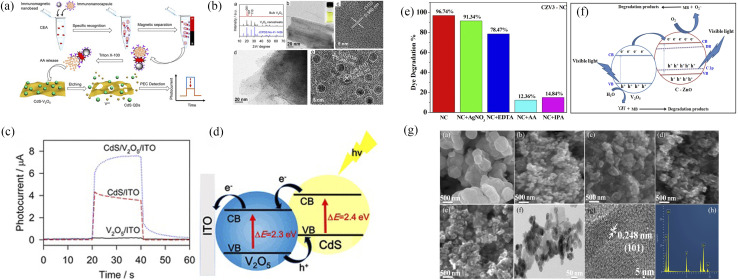
(a–d) Synthesis, morphology and photocatalytic degradation of V_2_O_5_–CdS (reproduced from ref. 30 with permission from [ACS], copyright [2022]). (e–g) Morphology and photocatalytic degradation of V_2_O_5_–ZnO (reproduced from ref. 32 with permission from [Springer], copyright [2022]).

In addition, there were alternative semiconductor materials to recombine with V_2_O_5_, such as CdS quantum dots,^30,31^ ZnO,^32^ and others (Fig. 12).

### Noble metal deposition

**Fig. 13:**
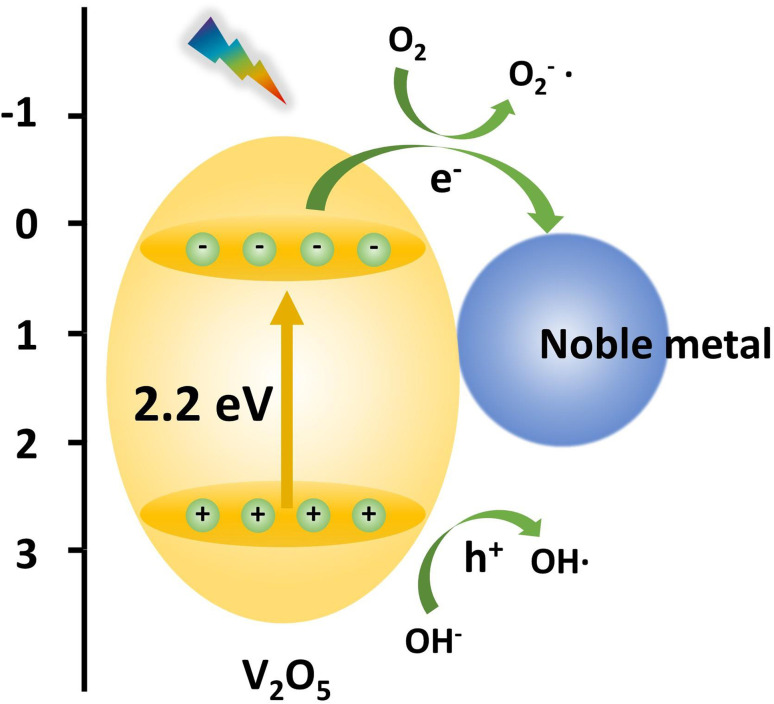
The scheme of the band structure of V_2_O_5_ and the noble metal.

**Fig. 14:**
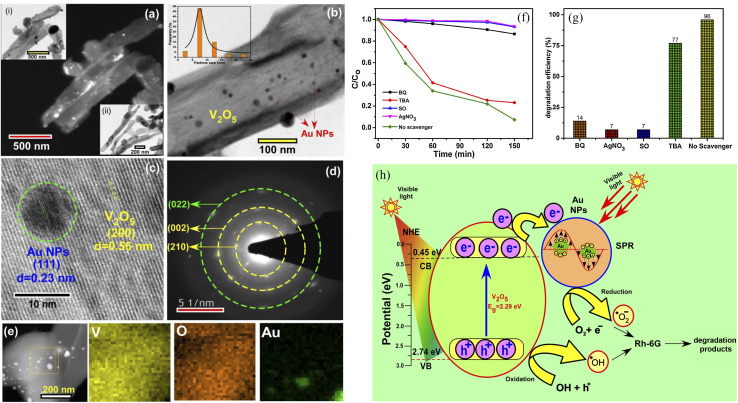
(a–e) Characterization, (f and g) photocatalytic degradation of R6G, (h) mechanism of Au-decorated V_2_O_5_ nanorods (reproduced from ref. 34 with permission from [Elsevier], copyright [2019]).

**Fig. 15:**
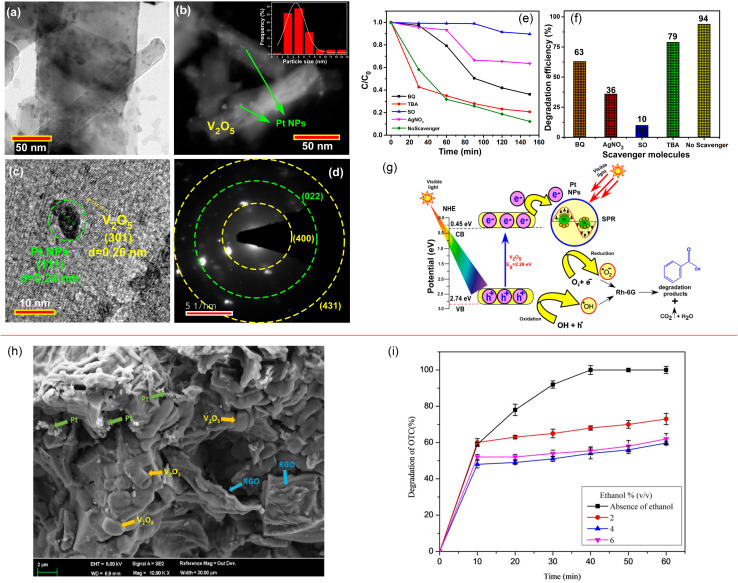
(a–g) Characterization, photocatalytic degradation and mechanism of Pt-decorated V_2_O_5_ nanorods (reproduced from ref. 35 with permission from [Elsevier], copyright [2020]). (h and i) Morphology and photocatalytic degradation of TOC of the Pt/V_2_O_5_ nanocomposites (reproduced from ref. 36 with permission from [Wiley], copyright [2020]).

The Fermi energy levels between noble metals and semiconductors are significantly different. When noble metals are deposited on the surface of semiconductors, photogenerated electrons will migrate from the semiconductors to the noble metals. Once the Fermi energy levels of the two are matched, the noble metals capture a large amount of negative charge, while the semiconductors obtain the corresponding positive charge, leading to the band bending and formation of the Schottky barrier, which can effectively prevent carrier recombination and improve the yield of photogenerated electrons (Fig. 13). Studies have shown that the photocatalytic activity of the composite materials modified by gold (Au), silver (Ag), platinum (Pt) and other noble metals is better than that of the photocatalytic materials prepared by pure semiconductor oxides. Adding an appropriate amount of noble metals can promote efficient electron–hole separation,^33^ thus improving the photocatalytic effect. Recently, considerable research efforts have been devoted to Au or Pt nanostructures for modification of photocatalytic materials due to the excellent stability and significant photocatalytic performance. For example, in Fig. 14, Au-decorated V_2_O_5_ nanorods were prepared through a one-step template-free hydrothermal method by Kumar's group. These nanorods exhibited a higher degradation of R6G dye when compared to pure V_2_O_5_.^34^ It was found that superoxide radicals O^2−^ and e^−^ were responsible for the enhanced photodegradation. Kumar *et al.* confirmed that Pt-decorated V_2_O_5_ nanorods also showed enhanced photocatalytic degradation of R6G dye under visible light irradiation, and the Pt NPs-assisted V_2_O_5_ increased the light response of the photocatalyst in the visible region with a maximum of 94% degradation with 1 wt% Pt,^35^ as shown in Fig. 15. Seralathan *et al.* also reported on the Pt/V_2_O_5_ nanocomposites for the efficient photocatalytic degradation of oxytetracycline.^36^

**Fig. 16:**
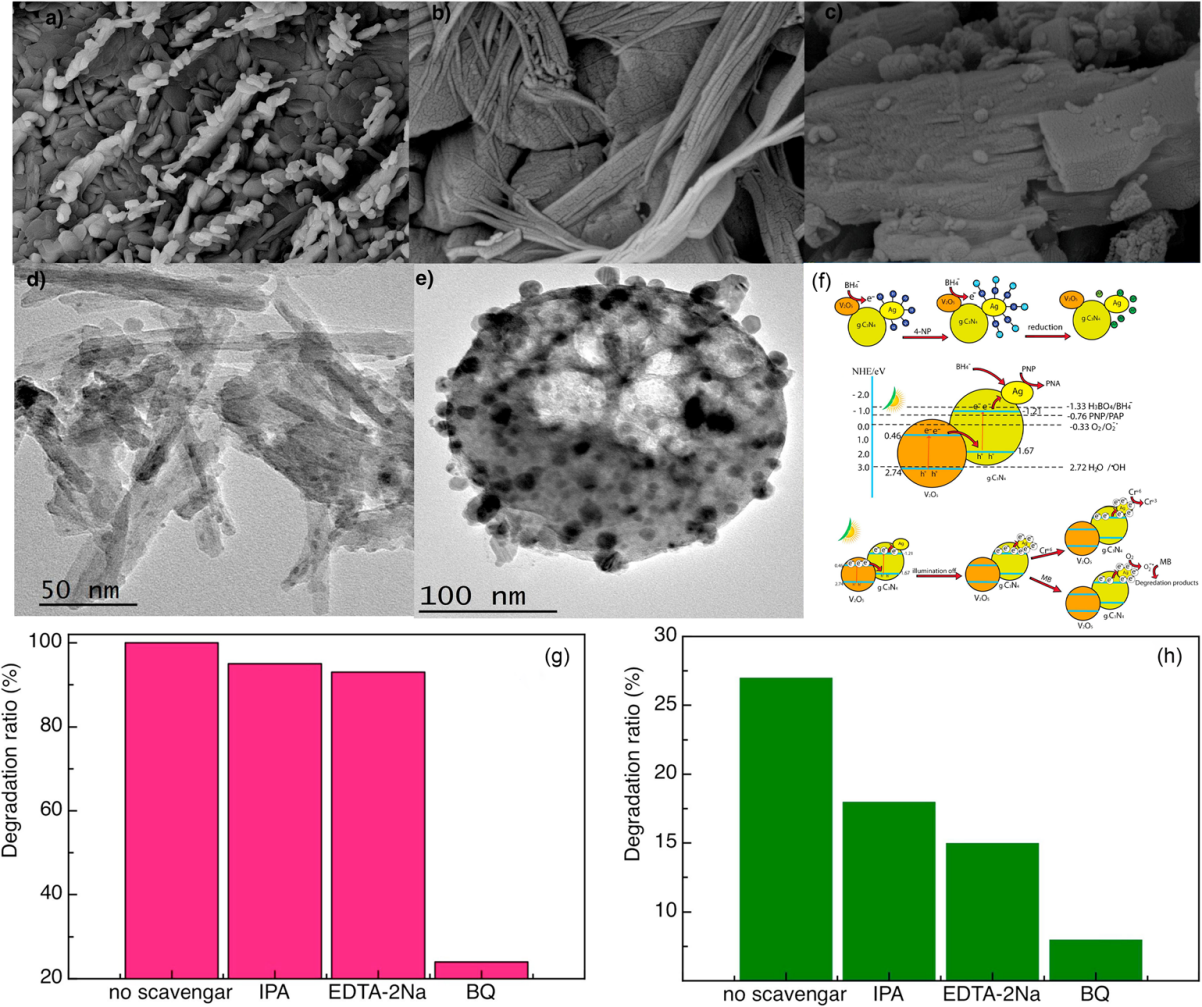
(a–e) Morphology, (f) mechanism, (g and h) photocatalytic degradation of the Ag/V_2_O_5_ nanocatalyst (reproduced from ref. 37 with permission from [Elsevier], copyright [2019]).

However, the expensive price and complex preparation process of gold or platinum nanomaterials limited their practical application and large-scale promotion. In contrast, Ag is lower priced than Au or Pt, and may have more potential for commercialization. As can be seen in Fig. 16, the Ag/V_2_O_5_ nanocatalyst prepared by Sheshtawy *et al.* exhibited unique catalytic, photocatalytic and post-oxidation/reduction ability for the removal of organic, *p*-nitrophenol, methylene blue and inorganic water pollutants.^37^ On the one hand, by depositing Ag nanoparticles on the surface of V_2_O_5_, the recombination of photogenerated electrons and holes was effectively inhibited by changing the electron energy level structure of the system. On the other hand, by depositing Ag nanoparticles on the surface of V_2_O_5_, the surface properties of V_2_O_5_ were changed and the photon yield of photocatalysis was improved. Moreover, the local surface plasmon resonance effect of Ag nanoparticles was used to enhance the visible light absorption capacity of the composites, and finally improved the photocatalytic performance of V_2_O_5_ system.

### Others

Several excellent reviews and research studies describing these multiple methods are available, and efficiently improved the photocatalytic performance of V_2_O_5_. For instance, the as-prepared Ag and ZnO co-doped V_2_O_5_ composites were employed in the photodegradation of Congo red dye under visible irradiation at ambient temperature,^38^ which took advantage of both semiconductor recombination and noble metal deposition to increase the photocatalytic activity of pure V_2_O_5_. The use of special materials, such as MXene, graphene, g-C_3_N_4_, black phosphorus, and others, was also used to overcome its own defects of V_2_O_5_. The Ti_3_C_2_ MXene/NiFe_2_O_4_/V_2_O_5_ ternary composites were constructed by Lam and Zeng *et al.*, which exhibited improved photoactivity for the decomposition of RhB, *Staphylococcus aureus* and *Bacillus cereus*.^39^

Application of V_2_O_5_ photocatalytic materials is not limited to the photodegradation of dyes. It is of great significance to explore the wide range of applications where V_2_O_5_ photocatalytic materials have been utilized, including pollutant degradation,^40^ water splitting,^41,42^ CO_2_ reduction,^43–45^ and organic synthesis,^46–48^ and others.

A variety of characterization techniques for V_2_O_5_ photocatalytic materials are beneficial to understanding the nature of V_2_O_5_ and developing the photocatalysts. For example, the crystal growth of V_2_O_5_ nanopowders prepared by sol–gel and hydrothermal methods was investigated by XRD, and further used with Scherrer's formula to obtain the particle size of V_2_O_5_ nanopowders.15
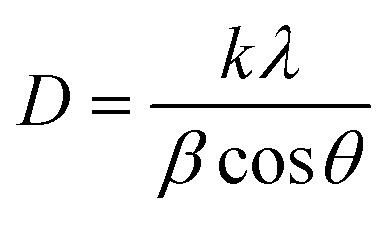


The average particle size of V_2_O_5_ nanopowders prepared by sol–gel method was calculated to be 290 nm, while the average particle size for hydrothermally prepared V_2_O_5_ nanopowders was 242 nm.^49^ Of course, in the sol–gel method, visual agglomeration of V_2_O_5_ particles was also found by SEM, which was not conducive to photocatalytic activity. Dizaji *et al.* also used XRD and SEM to study the graphene oxide-V_2_O_5_ nanocomposite prepared by a simple hydrothermal method.^50^ It was observed that the V_2_O_5_ crystalline phase began to appear when the powder was treated at 300 °C. Meanwhile, it presented a higher degree of crystallinity upon increase of the heat-treatment temperature to 400 and 500 °C, along with an increase of grain size, according to the Scherrer's equation. With the increase of calcination temperature, most of the V_2_O_5_ nanorods were converted to nanosheets, as seen from the SEM measurements. The specimen calcined at 400 °C was selected as the best one for preparing the nanocomposite in this work,^50^ which was confirmed by further photocatalytic properties. Sajid *et al.* also measured BET and obtained a nitrogen adsorption–desorption curve because the photocatalytic action was fundamentally related to the adsorption–degradation.^49^ The results indicated that the enhanced surface area and larger pore size of V_2_O_5_ by hydrothermal method provided new active sites for the facile transfer of the charge carrier and efficient adsorption of the reactant molecules,^49^ leading to superior photocatalytic activity with 99.1% and 82% degradation in 180 min for Congo Red and Methyl Orange, respectively. The UV-visible analysis revealed an energy band gap of 2.3 eV, which demonstrated that V_2_O_5_ possessed a great affinity for visible light absorption, and helped us understand the photocatalytic mechanism better.^49^ The optical band gap of the rGO–V_2_O_5_ nanocomposite was also studied by UV-vis, and calculated by use of Tauc's equation:^50^16
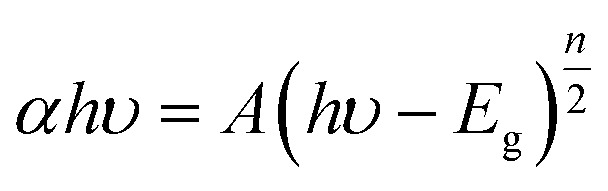


The absorption band gap of the V_2_O_5_ nanorod was about 2.26 eV, while the rGO–V_2_O_5_ nanocomposites exhibited a lower band gap of 1.60 eV, which indicated a higher absorbance of the nanocomposite compared with the V_2_O_5_ nanorods. As a result, the well-distributed rGO nanosheets and V_2_O_5_ nanorods showed a photodegradation efficiency of 85% in 255 min for the decolorization of the methylene blue dye.

## Conclusion and prospect

The modified vanadium pentoxide (V_2_O_5_) catalysts can be used for the selective and effective photocatalytic degradation of dyes, organic pollutants, poisonous gas and other substances. The modified V_2_O_5_ theoretically has excellent photocatalytic performance. However, V_2_O_5_ catalysts that are sufficiently stable and efficient for practical use have not yet been realized, and there are still some limitations in commercial application. With regard to doping, whether for metallic ion doping or non-metallic ion doping, it is inevitably subject to the influence of the type and concentration of doping ions. This brings difficulty in the preparation and regulation of structure, in order to introduce them into the V_2_O_5_ lattice and form the stable structure. Concerning semiconductor recombination, there is an urgent need for a perfect match between the valence and conduction potentials of the two semiconductors to form an effective structure. It is not easy for an appropriate choice of semiconductors to recombine with V_2_O_5_. Moreover, some semiconductors, despite their outstanding photocatalytic activity, are toxic and not environmentally friendly, such as CdS and WO_3_. Noble metal deposition has been proved to be a kind of efficient and simple means to improve the photocatalytic performance, but the high cost of noble metals is not friendly to the commercial photocatalyst. These special materials do not exhibit stable performance, and their role in the photocatalytic mechanism is not clear. Moreover, the unmarketable MXene, graphene and black phosphorus materials greatly increase the cost of research and development. Therefore, the research direction of V_2_O_5_ photocatalytic materials in the future should improve the photocatalytic efficiency, as well as reduce the cost.

On the one hand, two or more modification methods can be combined to promote the separation of photogenerated electrons and holes, improve the electron transfer rate, and thus enhance the photocatalytic efficiency. The combination of cheap semiconductors or metallic/non-metallic elements with noble metals may increase the photocatalytic activity of V_2_O_5_. On the premise of the effective improvement of the photocatalytic performance, it can also decrease the use of expensive noble metals.

On the other hand, during the process of modifying V_2_O_5_ photocatalytic materials in various ways, the reasonable design of composites needs further exploration and research, such as how to control the morphology and structure, as well as how to implement interface regulation.

Finally, V_2_O_5_ and V_2_O_5_-based composites are currently being extensively studied for the photodegradation of dyes, and have shown a relatively obvious photocatalytic effect. The following work should expand its application range, such as water splitting, organic synthesis, CO_2_ reduction, so as to be truly applied in practice.

## Conflicts of interest

There are no conflicts to declare.

## Supplementary Material
